# Hidden early-warning signals in scale-free networks

**DOI:** 10.1371/journal.pone.0189853

**Published:** 2017-12-18

**Authors:** Georg Jäger, Christian Hofer, Marie Kapeller, Manfred Füllsack

**Affiliations:** Institute of Systems Sciences, Innovation and Sustainability Research, University of Graz, Graz, Austria; Universitat Rovira i Virgili, SPAIN

## Abstract

Critical transitions of complex systems can often be predicted by so-called early-warning signals (EWS). In some cases, however, such signals cannot be detected although a critical transition is imminent. Observing a relation of EWS-detectability and the network topology in which the system is implemented, we simulate and investigate scale-free networks and identify which networks show, and which do not show EWS in the framework of a two state system that exhibits critical transitions. Additionally, we adapt our approach by examining the effective state of the system, rather than its natural state, and conclude that this transformation can reveal hidden EWS in networks where those signals are otherwise obscured by a complex topology.

## Introduction

Many systems in various scientific fields are known to exhibit so-called critical transitions, in which a system changes abruptly from one state into another. Prominent examples include climate change [1, 2], asthma attacks [3], epileptic seizures [4, 5], systemic market crashes [6] and catastrophic shifts in ecosystems [7].

For each of these systems, in particular in cases of transitions from beneficial to disadvantageous system states, it is of vital importance to accurately predict such critical transitions or at least find signals whether a critical transition is imminent or not [8]. One approach to making such predictions is looking for early-warning signals (EWS), i.e. various statistical indicators related to certain properties of the system [9].

However, not all systems that exhibit critical transitions show these EWS [10]. The investigation of EWS and the question in which systems they can and cannot be observed is a very active field, particularly in the context of networks. Especially in complex networks, scanning for EWS is very challenging. For example, the critical transition in a complex network might not correspond to a bifurcation [11]. However, there are network techniques that can be used to make detecting EWS easier, like considering spatial correlation in contrast to pure time series [12]. In order to monitor changes in this spatial correlation, an interaction network approach can be used [13]. Thus, the idea of including topological information in an EWS analysis is well established. However, most research regarding the combination of EWS and networks focuses on one specific network and it is difficult to generalize insights from these investigations to different networks. We are therefore interested in obtaining results that can be generalized to a whole category of networks, in our case the important class of scale-free networks.

In this paper we look at a simulated two-level system that is known to exhibit critical transitions and investigate it in implementation in different scale-free networks. The big advantage of such an approach, in comparison to other research in this field, is that we do not focus on one specific example for a network, like a bank network [14] or an ecosystem [12, 13], but we investigate randomly generated scale-free networks and simulate many such networks. This means that our findings are easier to generalize to all scale-free networks. Additionally we propose a simple way to include topology in EWS investigations. Normally this is a difficult process, but in this paper we condense all topological information, combined with the state of each node in the network, to one single scalar that can then be analyzed for EWS in the same way an ordinary time series is analyzed. A further advantage of our approach is that we are able to investigate scale-free networks with different power laws and compare the results. All differences in behavior we observe, can then be attributed to the different power law, since other parameters are fixed and details of the topology average out.

We find that in some topologies EWS can be detected; in others however, they remain hidden. Furthermore we explore a method to make these hidden EWS visible by transforming the system from its natural state to a so-called effective state, by a method which was recently suggested by Gao et al. [15].

This paper is organized as follows: Section 2 gives a short introduction to EWS. Section 3 introduces in the kind of scale-free networks examined. The example system we use for our simulations is explained in Section 4 and mathematical details on how to calculate the effective state of a system are given in Section 5. Numerical details of the simulation can be found in Section 6. Results are presented in Section 7 and discussed in Section 8.

### Early-warning signals

Systems theory describes dynamical systems as having an ‘eigen-behavior’ [16] which makes them evolve towards stable states. Internal interaction dynamics can cause systems to have several such stable states, spanning basins of attraction in the reach of which dynamics tend to return to after (small) perturbations [17]. The edges of these attractor basins (or potential wells) mark tipping points at which transitions to alternative stable states may occur. Depending on the strength of positive feedback effects, these transitions can be abrupt, showing sudden shifts of regime [18], so called ‘critical transitions’.

In respect to the suddenness of these shifts, attempts have been made to derive signals for the approach of a tipping point at which critical transitions occur. One such indicator is the time a system needs to return to equilibrium after perturbation. Slowing down of recovery time, so called ‘critical slowing down’ (CSD), has been found to indicate the loss of a system’s resilience and thus the approach of a transition [19].

CSD shows in the form of various statistical signals. One of them concerns changes in the correlation structure of a time series, caused by an increase in the ‘short-term memory’ (i.e. the correlation at low lags) of a system prior to a transition and is measured with autocorrelation at-lag-1 of consecutive observations [20]. Another indication for CSD is provided by the tendency of a system to drift more widely around its stable state when approaching a tipping point. This causes the standard deviation in the time series to increase. And finally, variance can show asymmetries and more extreme values when a system’s state gets close to the attraction of an alternative stable state. This causes skewness and kurtosis to change.

There are several case studies, where EWS are used for a variety of problems, such as predicting regime shifts in ecosystems [21], climate change [22] or land surface change [23].

### Scale-free networks

In a scale-free network the degree distribution follows a power law: The fraction of nodes with degree *k* is proportional to *k*^−*γ*^:

$$\begin{array}{c} P\left(k\right)∼k^{-\gamma } \end{array}$$ (1)

Scale-free networks are relatively common, for example social networks, the World Wide Web [24–26], the internet [27–29], citation networks [30], scientific collaboration networks [31–33] and metabolic networks [34] are scale-free. These networks have many intriguing properties: They contain so-called hubs, i.e. nodes with a degree much higher than the average degree of the network, their clustering coefficient distribution follows a power law, and they are very fault tolerant. [35–37]

In order to generate such a scale-free network we use a mechanism proposed by Goh et al. [38]. We start by generating *N* nodes and numbering them with *i* = 1, 2, …, *N*. Then we arbitrarily choose a control parameter *α* in the interval [0, 1). Each node is then assigned a connection probability *p*_*i*_ = *i*^−*α*^. Next we select two nodes *a* and *b* with probabilities equal to *p*_*a*_/∑_*i*_ *p*_*i*_ and *p*_*b*_/∑_*i*_ *p*_*i*_ respectively. If those two nodes are not already connected, a link is established. This process is repeated, until a previously determined number of links is reached. Since the links are distributed according to this probability, it can be calculated that the degree of the nodes obeys the power law [38]

$$\begin{array}{c} P\left(k\right)∼k^{-\gamma } \end{array}$$ (2)

where *γ* is closely related to the control parameter *α* via

$$\begin{array}{c} \gamma =(1+\alpha )/\alpha \end{array}$$ (3)

That way it is possible to generate a scale-free network with well-defined *γ* ≥ 2 by choosing *α* appropriately.

## Methods

### A two state system

In order to gain universal results, not depending on the specifics of a certain system, we investigate a simple two state system, consisting of a network of *N* nodes and *L* links, connecting these nodes. Each node can be in one of two different states, denoted as ‘state 1’ and ‘state 2’. The meaning of these states depends on the investigated system. For a cooperative system of human beings the states could represent ‘cooperating’ and ‘not cooperating’, for an ecosystem comprised of different species that live in symbiosis the states could represent ‘not endangered’ and ‘endangered’ and many more interpretations are possible.

We can generalize our results to all systems that have the following properties: All nodes initially start in state 1 and have a gradually increasing chance to change to state 2. Neighboring nodes can, however, transfer a node in state 2 back to state 1. This mixture of positive and negative feedback-loops guarantees a critical transition and is a valid approximation to many real-life systems. Details on the system are given in section 6.

### The effective state of a system

The natural state of a system (in our system this corresponds to the number of nodes in state 1) can be used to scan for EWS and for many systems this approach is well-established [39–41]. However, it is equally possible to calculate the so-called *effective state* of a system and use this property to scan for EWS. Calculating effective states was proposed by Gao et al. [15] in order to derive a one-dimensional dynamic from a complex multi-dimensional system. There, effective parameters are calculated as

$$\begin{array}{c} \frac{I^{T}\;A\;x}{I^{T}\;A\;I} \end{array}$$ (4)

with the adjacency matrix *A*, the unit vector *I*, and *x*, the investigated property of the system (in our case *x* = 1 for nodes in state 1 and *x* = 0 for nodes in state 2). This operation corresponds to taking an average over the whole network, where each node is weighted with its degree, i.e. the number of links attached to it. For networks where all nodes have the same degree, the effective state and the natural state are equivalent. The difference becomes more important for networks where some nodes are very well connected, while others are not. Take a very simple network of 4 nodes, where one node serves as a hub and is connected to all other nodes, while there are no other links in the system. Suppose the hub is in state 1, all other nodes are in state 2. The natural state of the system, scaled to the number of nodes can then be calculated as

$$\begin{array}{c} \frac{N^{\left(1\right)}}{N}=\frac{\left(1+0+0+0\right)}{4}=0.25 \end{array}$$ (5)

The interpretation of this property is that 25% of all nodes are in state 1, i.e. if you select one node of the system at random, the chance that it is in state 1 is 25%. However, the fact that the central hub of this network, the node with the most influence on the system, is the one in state 1, is neglected in this simple average. As long as exactly one node is in state 1, the natural state is 0.25. This is different for the effective state. Using the same example, we can calculate

$$\begin{array}{ccc} \frac{N_{\text{eff}}^{\left(1\right)}}{N} & = & {\left(\begin{array}{c} 1\\ 1\\ 1\\ 1 \end{array}\right)}^{T}\left(\begin{array}{cccc} 0 & 1 & 1 & 1\\ 1 & 0 & 0 & 0\\ 1 & 0 & 0 & 0\\ 1 & 0 & 0 & 0 \end{array}\right)\left(\begin{array}{c} 1\\ 0\\ 0\\ 0 \end{array}\right)*{\left({\left(\begin{array}{c} 1\\ 1\\ 1\\ 1 \end{array}\right)}^{T}\left(\begin{array}{cccc} 0 & 1 & 1 & 1\\ 1 & 0 & 0 & 0\\ 1 & 0 & 0 & 0\\ 1 & 0 & 0 & 0 \end{array}\right)\left(\begin{array}{c} 1\\ 1\\ 1\\ 1 \end{array}\right)\right)}^{-1}\\ & = & {\left(\begin{array}{c} 1\\ 1\\ 1\\ 1 \end{array}\right)}^{T}\left(\begin{array}{c} 0\\ 1\\ 1\\ 1 \end{array}\right)*{\left({\left(\begin{array}{c} 1\\ 1\\ 1\\ 1 \end{array}\right)}^{T}\left(\begin{array}{c} 3\\ 1\\ 1\\ 1 \end{array}\right)\right)}^{-1}\\ & = & 3*6^{-1}=0.5 \end{array}$$ (6)

The interpretation of this number is not straightforward, because it does not mean that 50% of the nodes are in state 1. However, if you select a random link from the system and then select one of the two connected nodes at random, the chance that this selected node is in state 1 is 50%. $N_{\text{eff}}^{\left(1\right)}$ is thus related to the chance to select a node in state 1 with a selection process that favors very well connected nodes, to be precise, all selection processes where the selection chance of each node is directly proportional to the degree of the node.

In a way the effective state of the system takes the different importance of each node into account, in contrast to the natural state, where this information is completely lost. It is therefore intuitive that the effective state of the system offers more insight into the particularities of the system and should hence be used to scan for EWS, especially for very complex network topologies.

### Simulation

The numerical details of our simulations are as follows: We use a population of *N* = 500, connected via *L* = 2500 links. The chance of a node switching from state 1 to state 2 begins at *s*_12_ = 0 percent and is increased by *ds*_12_ = 0.0002 percent each time step. The chance to switch back to state 1, *s*_21_, is dependent on the link-neighbors: Each link-neighbor in state 1 gives a node in state 2 a 1 percent chance to switch back to state 1. Note that the actual switch-chances have no influence on the qualitative behavior of the system, they would only translate the time of the critical transition. Different values of *ds*_12_ and *s*_21_ were tested. The only qualitatively different behavior is observed, if *s*_21_ is too small to give the system any resilience, in which case state 1 is not stable and all nodes reach state 2 nearly instantly.

In our simulations we analyze the system detailed above by counting the nodes in state 1 in each time step to find *N*^(1)^, the number of nodes in state 1. Additionally we calculate $N_{\text{eff}}^{\left(1\right)}$, the effective number of nodes in state 1 according to Eq (4).

**Fig 1:**
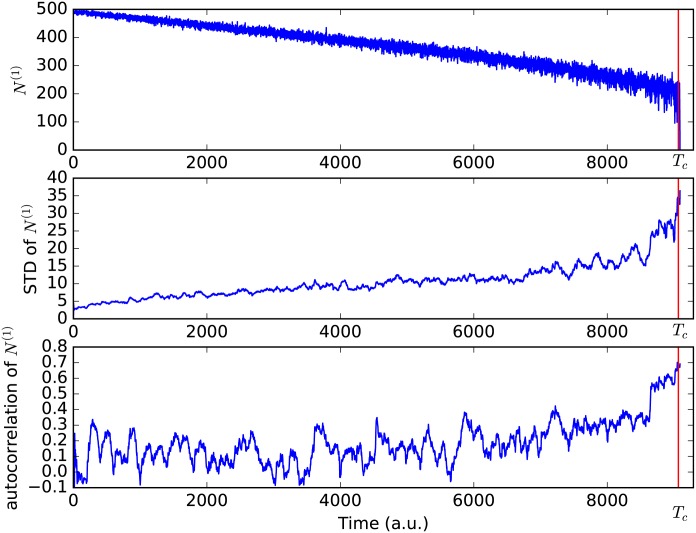
Typical time development of the number of nodes in state 1. *N*^(1)^ decreases over time, until there is a critical transition at *t* = *T*_*c*_ (red vertical line). When looking at the standard deviation and the autocorrelation of *N*^(1)^, one can see that it increases significantly prior to the critical transition, which constitutes an EWS.

A typical time development of the natural state of such a system is shown in Fig 1: *N*^(1)^ decreases until it reaches 0 after a critical transition. The constant increase of standard deviation is a property of the system; it is, however, also clearly visible, that the standard deviation of *N*^(1)^ increases sharply right before the critical transition. In order to verify that this can indeed be seen as an EWS we also need to investigate the autocorrelation, depicted in the lower panel of Fig 1. Here we can see that the autocorrelation increases significantly before the critical transition as well.

**Fig 2:**
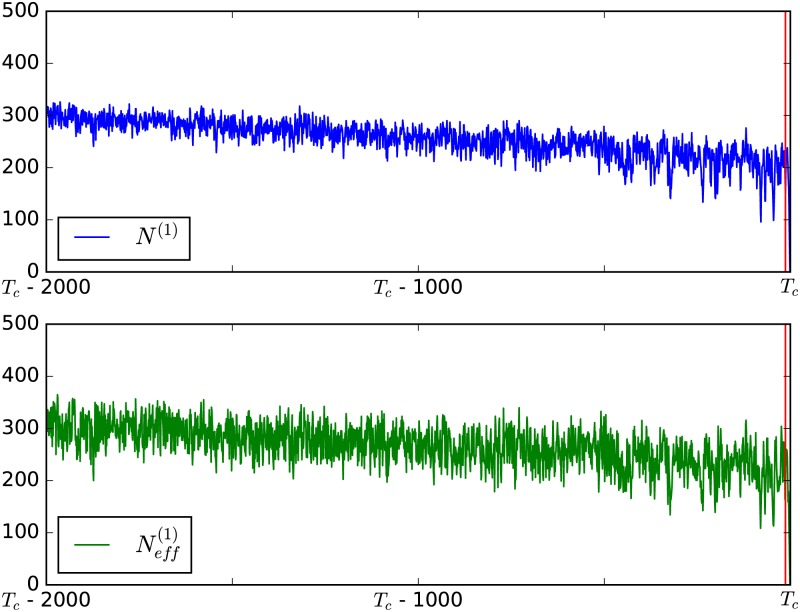
Natural and effective state of the system. *N*^(1)^ and $N_{\text{eff}}^{\left(1\right)}$ behave similarly, however, the effective state has a higher variance.

The difference between the effective and the natural state is illustrated in Fig 2. Both serve as a measurement of what fraction of the system is in state 1, yet the particularities concerning the individual importance of each node, detailed above, lead to a slight difference, most prominently visible in a higher variance of $N_{\text{eff}}^{\left(1\right)}$.

In order to scan for EWS we analyze the standard deviation and the autocorrelation of both *N*^(1)^ and $N_{\text{eff}}^{\left(1\right)}$ in a rolling window of 100 time steps. In order to constitute an EWS, both standard deviation and autocorrelation must increase before the critical transition. We simulate scale-free networks with *α* values of 0.125, 0.25, 0.5, and 1 corresponding to *γ* values of 9, 5, 3, and 2 respectively.

## Results

**Fig 3:**
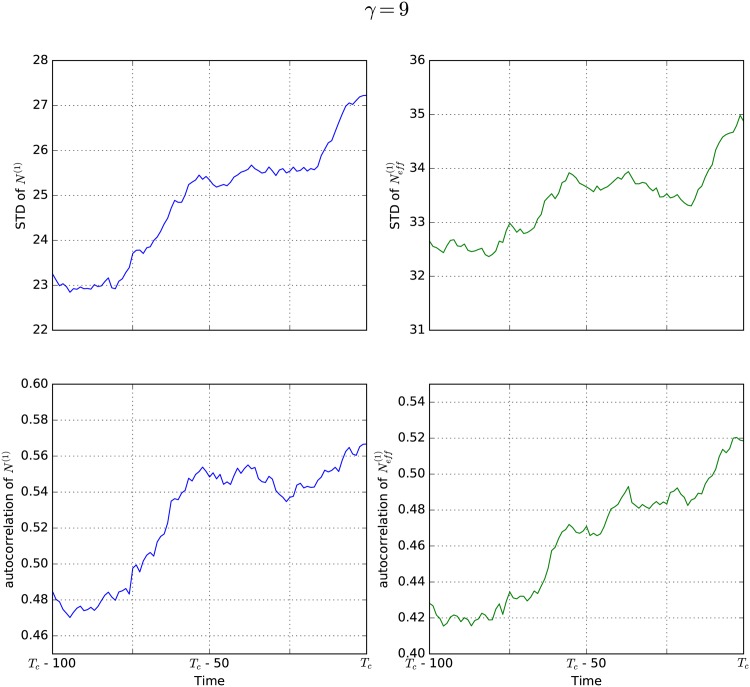
EWS for *γ* = 9. An increase in standard deviation as well as autocorrelation can be observed prior to the critical transition, both for the natural state *N*^(1)^ (blue) and the effective state $N_{eff}^{\left(1\right)}$ (green) in networks with *γ* = 9.

**Fig 4:**
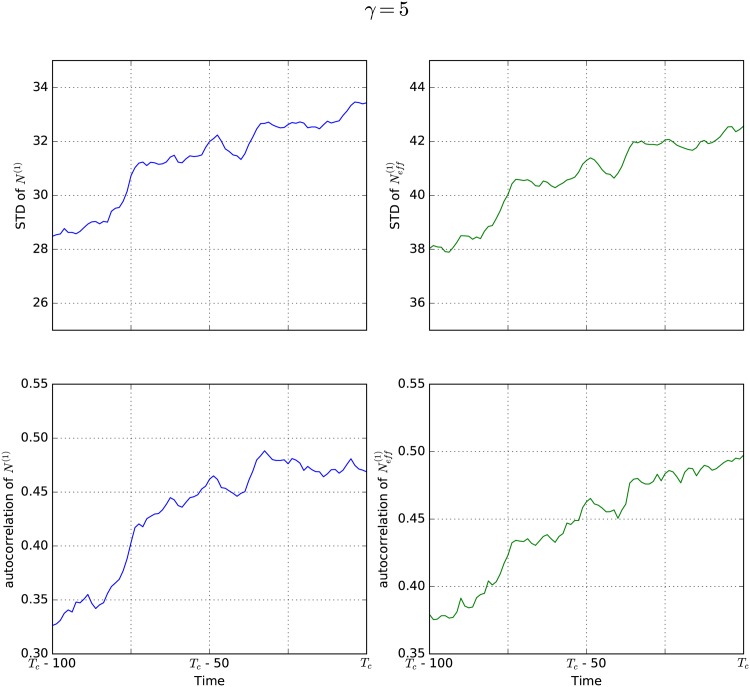
EWS for *γ* = 5. An increase in standard deviation as well as autocorrelation can be observed prior to the critical transition, both for the natural state *N*^(1)^ (blue) and the effective state $N_{eff}^{\left(1\right)}$ (green) in networks with *γ* = 5.

**Fig 5:**
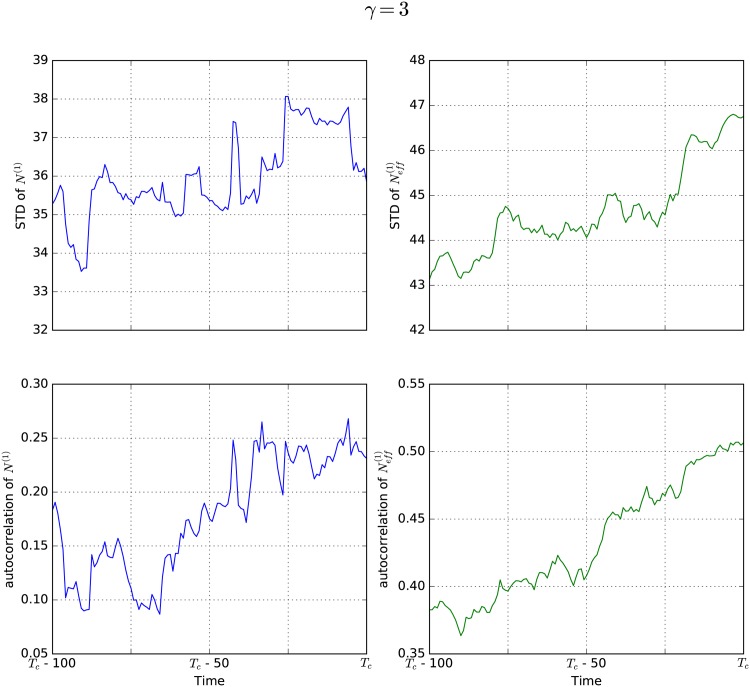
EWS for *γ* = 3. An increase in standard deviation as well as autocorrelation can be observed prior to the critical transition, both for the natural state *N*^(1)^ (blue) and the effective state $N_{eff}^{\left(1\right)}$ (green) in networks with *γ* = 3. Note that the effective state shows a much clearer signal with less fluctuations than the natural state.

**Fig 6:**
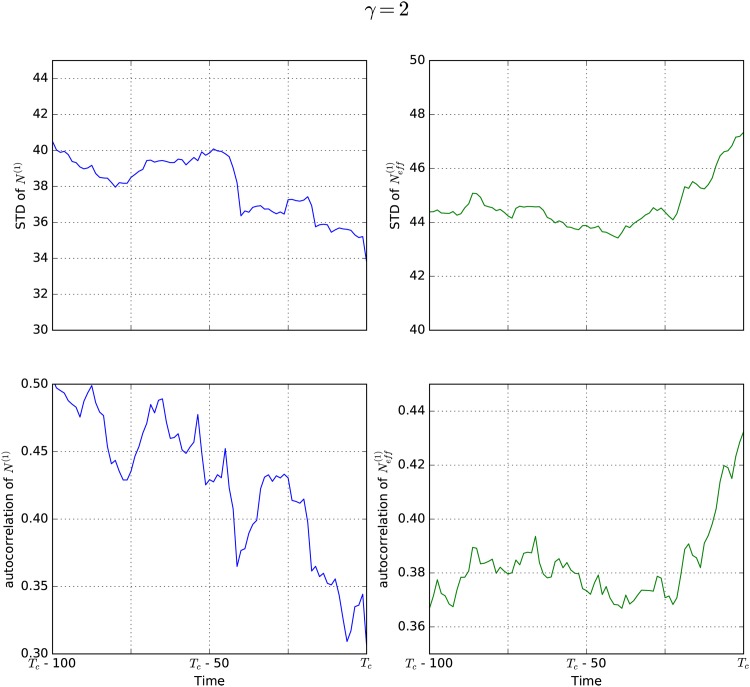
EWS for *γ* = 2. While the natural state of the system *N*^(1)^ (blue) shows no EWS (both standard deviation and autocorrelation decrease), the EWS are clearly visible for the effective state $N_{eff}^{\left(1\right)}$ (green) in networks with *γ* = 2.

The results of our simulations are reported in Figs 3–6. The left side of each figure (blue) shows standard deviation (upper panel) and autocorrelation (lower panel) for the natural state *N*^(1)^. The right side (green) shows the same properties for the effective state $N_{\text{eff}}^{\left(1\right)}$. For each value of *γ* the results were obtained by averaging over simulations of 10 different networks in order to rule out that the observed effects are random features of a specific network, but rather features of all scale-free networks with this *γ* value. Different networks of course lead to different times for the critical transition *T*_*c*_, so in order to average over them it is necessary to measure time relative to *T*_*c*_. As indicated on the x-axes, we plot all values against the difference between the time *T*_*c*_ and the time *t*.

Fig 3 shows the resulting EWS for scale-free networks with *γ* = 9. In these networks we can observe an increase in standard deviation as well as autocorrelation for both the natural state (blue) as well as for the effective state (green). Here, effective and natural state are very similar, since a large fraction of the nodes has a degree very close to the average degree. Therefore, also standard deviation and autocorrelation of *N*^(1)^ and $N_{\text{eff}}^{\left(1\right)}$ do not differ much.

Fig 4 presents our simulation results for scale-free networks with *γ* = 5. Again, standard deviation as well as autocorrelation increase before the critical transition at *t* = *T*_*c*_. This effect can be seen in the natural state (blue) as well as in the effective state (green).

Fig 5 displays results for scale-free networks with *γ* = 3. Here, there is a visible difference between investigating the natural state of the system (blue) and the effective state of the system (green). While both properties lead to an EWS, i.e. an increase in both standard deviation and autocorrelation, the signal is much clearer for $N_{eff}^{\left(1\right)}$. In these networks, not all nodes are equally well connected. Some nodes have very few links, while others have a very high degree, which gives them more influence on the total state of the system. Neglecting these differences by investigating the natural state leads to a less distinct, but still visible EWS.

Fig 6 reports the findings of our simulations for scale-free networks with *γ* = 2. Here, the results are qualitatively different from those obtained for networks with *γ* ≥ 3. When investigating the natural state of the system (blue), no EWS can be detected. Both standard deviation and autocorrelation even decrease before *T*_*c*_. However, when looking at the effective state of the system (green) the EWS are still clearly visible. The huge difference between the behavior of *N*^(1)^ and $N_{\text{eff}}^{\left(1\right)}$ can be attributed to the network topology, specifically to the parameter *γ*, since it is the only difference to the other simulations.

## Discussion

We conclude that analyzing a scale-free network for EWS is not straightforward, especially when considering networks with *γ* ≤ 3. In our example system we observe the standard deviation and the autocorrelation of both *N*^(1)^, the natural state of the system, as well as $N_{\text{eff}}^{\left(1\right)}$, the effective state of the system. In scale-free networks with *γ* < 3 we are able to detect clear signals both for the natural as well as for the effective state. For scale-free networks with *γ* = 3 the signals are visible for both *N*^(1)^ and $N_{\text{eff}}^{\left(1\right)}$, however they were clearer for the effective state. For *γ* = 2 the situation is different. Scanning for EWS in the natural state of the system reveals no EWS; both standard deviation and autocorrelation even decrease shortly before the critical transition. Nevertheless, analyzing the effective state of the system does produce EWS. Since the only difference between the investigated systems is the parameter *γ*, we deduce that the exponent of the power law for the scale-free network is responsible for this qualitative difference. The same system, implemented in different networks, can show EWS in one case, while showing none in the other case, even though there is a critical transition in both topologies. This finding substantiates that not only the interaction between the nodes of a network, but also the topology itself influences, if EWS are detectable or not. Especially when investigating networks with well connected hubs it is therefore paramount to include topological effects when scanning for EWS, possibly by using the effective state of a system, rather than its natural one.

In scale-free networks with a small *γ* parameter the degree distribution is far from uniform. There are very big hubs, i.e. nodes that have many links and are therefore very important for the development of the system as a whole. Nodes that have only few connections have less influence. When investigating the natural state of the system we completely ignore these differences and scanning for EWS yields no results, since this implicitly assumes that the sum of nodes in one state is a good measurement for the overall state of the system. This assumption is valid for scale-free networks with high *γ*, but it is questionable for networks where the degree distribution is far from uniform. If we want to include the difference in importance of the individual nodes we have to investigate the effective state of a system, rather than its natural state.

In conclusion, our investigations show that there are systems in which one cannot find EWS, although a critical transition is imminent, when only considering the natural state of the system. Analyzing the effective state of the system can reveal these hidden EWS. Our research suggests that the benefit of using the effective state of a system rather than its natural one is greater, the less uniform the links are distributed. It is likely, that the possibility for hidden EWS is not only a property of scale-free networks, but rather of all networks, where the degree distribution is far away from uniform. To illuminate this, further research is required and should focus on investigating different systems that show critical transitions and different types of networks beyond the scope of scale-free ones in order to find universal insights into the connection between topology and EWS detectability.
